# An Unidentified Infiltrative Etiology of Spinal Cord Compression: A Case Report and Literature Review

**DOI:** 10.7759/cureus.60141

**Published:** 2024-05-12

**Authors:** Michael J Valentine, Fakhar Hayat, Ankur Kayastha, Takara R Newsome-Cuby, Anh Thu N Nguyen, Usama AlDallal, Mohamed Ismail

**Affiliations:** 1 Medical School, Kansas City University, Kansas City, USA; 2 Neurosurgery, King Hamad University Hospital, Al Sayh, BHR; 3 School of Medicine, Royal College of Surgeons In Ireland - Medical University of Bahrain, Manama, BHR

**Keywords:** neurosurgery, back pain, langerhans cell histiocytosis, non-neoplastic mass, case report, spinal cord compression

## Abstract

Spinal cord compression is a neurosurgical emergency. Symptoms of this disorder are highlighted as back pain, ambulatory difficulties, and bladder/bowel incontinence. Diagnostic imaging is not indicated in many circumstances of nonspecific back pain; however, the addition of neurologic deficits in the setting of back pain justifies radiologic imaging. Various pathologies can cause constriction of the spinal cord due to the delicate nature of spinal cord anatomy. Etiologies may include trauma, neoplasms, and infections. In this report, we present an unusual case of a 31-year-old male who presented to the emergency department with a history of chronic back pain accompanied by neurological deficits, ataxia, and bladder dysfunction. Contrast-enhanced MRI imaging heightened the suspicion of a neoplastic etiology; however, neuropathology revealed a non-neoplastic nature with abnormal lymphohistiocytic infiltrate suspicious for Langerhans cell histiocytosis or infectious etiology. A second opinion was provided by Mayo Clinic Laboratories, resulting in the definitive conclusion that the mass was non-neoplastic and tested negative for SD1a and Langerhin, biomarkers used to diagnose Langerhans cell histiocytosis. This unusual non-neoplastic lesion exemplifies one of many diverse and multifaceted pathologies that can precipitate spinal cord compression. Additionally, these findings underscore the importance of considering both neoplastic and non-neoplastic causes in the differential diagnosis of spinal cord compression, thereby enhancing clinical vigilance and improving patient outcomes for underlying spinal conditions.

## Introduction

Spinal cord compression, characterized by damage or constriction of the spinal cord at any level within its encasement, can result from a multitude of etiologies impacting the structural integrity of the spinal column. Etiology is vast but may be categorized into traumatic or non-traumatic subtypes. Atraumatic causes notably include congenital factors, infectious origin, and primary or metastatic malignancy. Atraumatic etiologies occur less frequently than traumatic etiology [1]. For example, in the United States (US), the prevalence of malignant spinal cord compression among cancer patients was approximately 3.4% in 2011 [2]. The incidence of spinal abscesses has been experiencing an upward trend. This is partially attributable to the potential exposure of the spinal cord to bacteria from the increased frequency of spinal surgeries [3]. Furthermore, spinal abscess incidence is estimated to be 5.1 cases per 10,000 hospital admissions [4]. 

Clinically, spinal cord compression generally manifests with bilateral symptoms and presents with a variable onset that may be abrupt or gradual. Back pain is the most common initial symptom [1,5,6]. Motor deficits are often observed as bilateral paralysis at and below the level of spinal cord involvement, in addition to hyperreflexia and ataxia. Physical examination frequently reveals a positive Babinski sign. Sensory impairments, although variable, occur below the level of the affected spinal segment. Sphincter dysfunctions, such as urinary and bowel urgency, retention, or incontinence, are also notable clinical features of spinal cord compression. Treatment options depend on etiology and severity but may consist of radiotherapy and/or surgical intervention [7-9].

In this report, we present the case of a 31-year-old male with spinal cord compression secondary to an unusual non-neoplastic mass containing histiocytic and lymphoplasmacytic infiltrate. The lesion bore a resemblance to Langerhans cell histiocytosis but was negative for CD1a. Given the abnormal nature of this presentation, pathology specimens were sent to Mayo Clinic Laboratories for a second opinion, which corroborated the non-neoplastic characterization of the lesion. This case underscores the vast variability of pathologies causing mass effects and subsequent spinal cord compression.

## Case presentation

Patient presentation

A 31-year-old male presented to the emergency department for a neurosurgical consultation. He had a history of chronic back pain accompanied by progressive weakness in both lower limbs, unsteadiness, and ataxia that had been developing over the preceding two months. His back pain dates back to 2018. He reported a gradual exacerbation of weakness over the past two weeks, culminating in reliance on crutches for ambulatory support. This decline in physical capability had adversely impacted his daily activities, as evidenced by difficulty with micturition and increased constipation. Moreover, he also reported experiencing bilateral leg numbness for the past 7-10 days. His condition had deteriorated in the three days prior to presentation, leading to an inability to stand or walk without substantial assistance. He denied any recent episodes of fever, loss of consciousness, or headaches. 

Diagnostic evaluation

During the clinical examination, the patient was found to be alert, oriented, and hemodynamically stable. He exhibited intact sensation in the upper limbs but severe bilateral paraparesis in the lower limbs. An assessment of active range of motion revealed extensive impairment of lower extremity movement bilaterally. Special and normal sensory examination was intact up to the T10 level; however, he demonstrated marked perianal anesthesia and significantly reduced anal tonicity.

**Figure 1:**
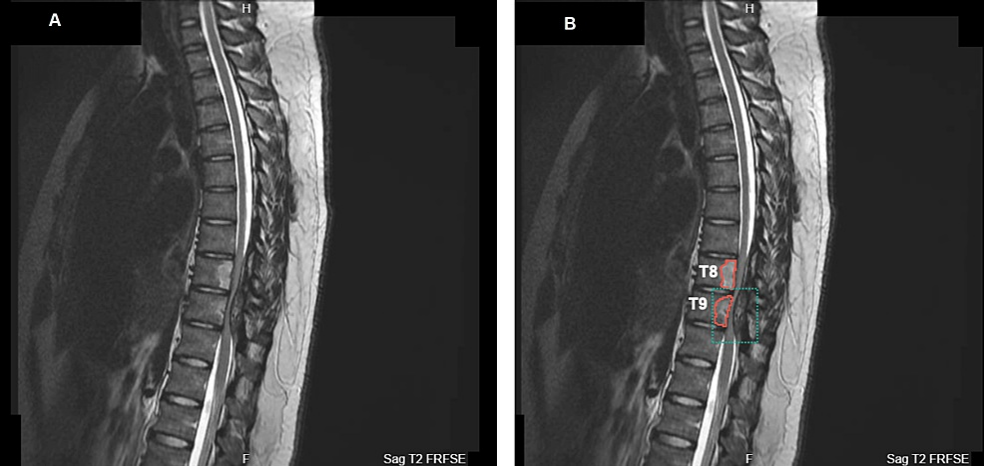
Sagittal T2 weighted fast recovery fast spin echo demonstrating spinal cord compression (green dotted outline) at T8 and T9 levels with relief at T10. The pathology is highlighted in (B) as compared to (A). Additional findings show an epidural lesion suggestive of myelomalacia and positive bone marrow edema (red solid outline) findings. T8: thoracic vertebral segment 8; T9: thoracic vertebral segment 9; T10: thoracic vertebral segment 10

**Figure 2:**
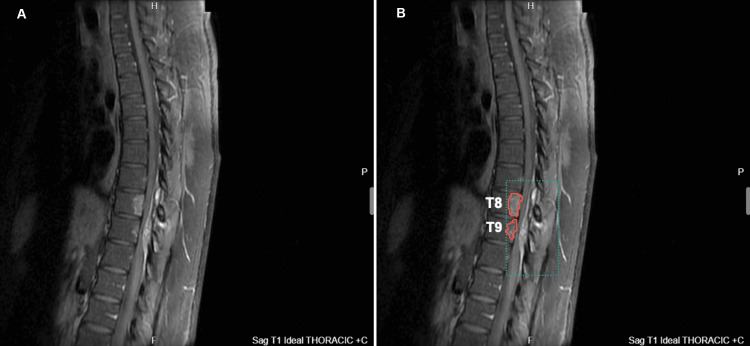
Sagittal T1 weighted image with contrast demonstrating spinal cord compression (green dotted outline) at T8 and T9 levels. The pathology is highlighted in (B) as compared to (A). Additional findings show an epidural lesion suggestive of myelomalacia and positive bone marrow edema (red solid outline) findings. T8: thoracic vertebral segment 8; T9: thoracic vertebral segment 9

**Figure 3:**
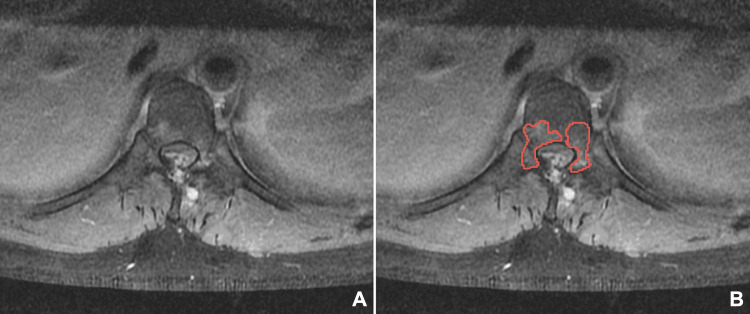
Axial T1 weighted contrast-enhanced MRI image demonstrating hyperintense bone marrow edema (red solid outline) within the vertebrae and spinal cord compression. The pathology is highlighted (B) as compared to (A).

An initial magnetic resonance imaging (MRI) scan revealed spinal cord compression, characterized by a posterior epidural lesion consistent with myelomalacia, as well as thoracic vertebral bone marrow edema at the T8 and T9 levels (Figures 1-3). Axial MRI sections depicted intervertebral edema within the epidural lesion with tubular extension towards the posterior paraspinal region (Figure 3), thus raising suspicions of vascular involvement and neoplasm.

Management and outcome

**Figure 4:**
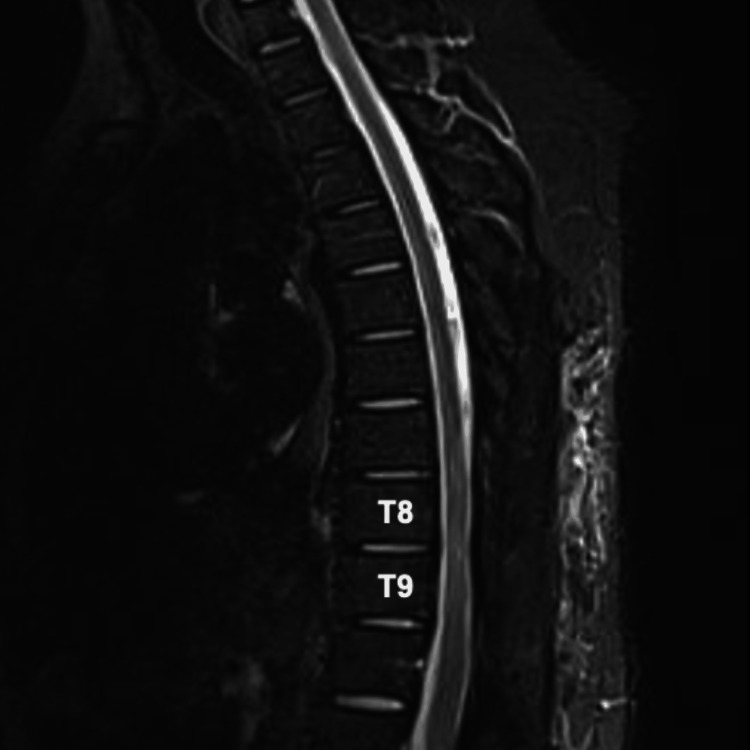
Postoperative T2 weighted sagittal MRI image demonstrating amelioration of spinal cord compression, epidural lesion, and spinal cord edema at the T8 and T9 levels. T8: thoracic vertebral segment 8; T9: thoracic vertebral segment 9

Following these clinical findings corroborated with imaging, the patient underwent an urgent, high-risk laminectomy along with thoracic spinal cord decompression and excision of the lesion. During the surgery, the lesion was observed to possess dense, fibrous, and multilayered characteristics. The lesion was exhibiting reactive changes within the surrounding cortical bone. Tissue samples, including both soft tissue and bone, were extracted for neuropathological examination. A follow-up MRI conducted postoperatively revealed a significant reduction in spinal cord compression and edema, revealing successful resection of the lesion responsible for his accompanying symptoms (Figure 4). 

Pathophysiology

The initial pathological analysis identified the soft tissue lesion as possibly being a dermoid cyst, an inclusion cyst, or a congenital cyst. Subsequent detailed examination in the pathology report confirmed the lesion as epidural and fibrous, noting its composition of both extraspinal and intraspinal elements. Microscopic examination of the bone fragments revealed unusual focused histiocytic infiltration accompanied by a lymphoplasmacytic infiltrate. Notably, the pathological analysis did not detect any epithelial elements within the bone samples, nor were there indications of cellular atypia.

Immunohistochemical testing showed that the histiocytic cells were positive for S100 and CD68, but negative for CD1a and Langerin biomarkers, ruling out Langerhans cell histiocytosis as a potential diagnosis. Additionally, these cells did not exhibit reactivity for AE1/3, CD20, SMA, Pax5, CD30, CD15, Mum1, CD34, desmin, and CD3 biomarkers. Following an internal review within the pathology department, a decision was made to seek a second opinion. Additional verification was needed to exclude the possibility of Langerhans cell histiocytosis and confirm the non-neoplastic nature of the lesion, including the negative CD1a and Langerin results. CD1a and Langerin negativity could have been influenced by prolonged decalcification affecting immunoreactivity. The conclusive diagnosis from Mayo Clinic Laboratories corroborated the non-neoplastic character of the biopsy specimens. The findings highlighted a reactive lymphohistiocytic infiltrate with acute inflammation, set within epidural fibroadipose tissue amongst fragments of bone and skeletal muscle. Key findings and a physical examination summary can be found in Table 1 and Table 2, respectively.

**Table 1:** Summary of the key findings of the case S100: a protein family that's soluble in 100% ammonium sulphate at a neutral pH; CD: cluster of differentiation; AE1/3: antibody cocktail used to indicate carcinomas; SMA: spinal muscular atrophy; Pax5: a B-cell specific activator protein; Mum1: multiple myeloma oncogene-1

Key Findings	
Presenting Symptoms	Chronic back pain with progressive weakness in bilateral lower limbs.
Review of Systems	Denied fever, loss of consciousness, and headaches.
Red Flags	Progressive weakness with recent rapid deterioration, nonambulatory, young age, bilateral leg numbness, micturition difficulty, increased constipation, and loss of sensation below T10.
Imaging	Contrast-enhanced MRI of the spine.
Diagnosis	Spinal cord compression secondary to unknown mass lesion.
Treatment	Laminectomy and thoracic spinal cord decompression with lesion excision.
In-House Pathology Results	Unknown etiology, non-neoplastic cyst with mixed lymphoplasmacytic and histiocytic infiltrate.
Mayo Clinic Pathology Results	Unknown etiology, non-neoplastic cyst with mixed lymphoplasmacytic and histiocytic infiltrate.
(+) Biomarkers	S100 and CD68.
(-) Biomarkers	AE1/3, CD20, SMA, Pax5, CD30, CD15, Mum1, CD34, CD3, Desmin, Langerin, and CD1a.

**Table 2:** Summary of the physical findings of the case T: thoracic level

Physical Examination	Normal or Abnormal	Findings
Alert and Oriented Status	Normal	Person, place, time, and event.
Hemodynamic Status	Normal	Stable, no headaches.
Active Range of Motion	Abnormal	Markedly decreased in lower limbs bilaterally.
Sensory Examination	Abnormal	Special and normal sensory intact to T10 level, marked perianal anesthesia and reduced anal tonicity, bilateral leg numbness.
Ambulatory Status	Abnormal	Ataxia, reliance on crutches for ambulation.

Follow-up

Following surgery, the patient was observed closely as he was a substantial fall risk. He was placed on bed physiotherapy, which he was able to tolerate three times daily. He continued oral feeding and a regular diet as tolerated. Postoperative medications included prophylactic vancomycin, dexamethasone, omeprazole, and enoxaparin. Wound dressings were changed daily until he was stable for discharge.

## Discussion

Back pain as a predominant indicator

The most common and earliest presenting symptom of spinal cord compression is back pain [1,5,6]. Considering the global prevalence of back pain as a primary cause of disability, it is anticipated that a significant portion of the adult population will experience back pain at some point in their lives [10,11]. The economic burden of back pain is substantial. One study estimates that the total healthcare expenditure for low back and neck pain in the US was $87.6 billion in 2013 [12]. This financial burden can be partly attributed to the early employment of diagnostic imaging, which may yield extraneous results, precipitating unnecessary and costly subsequent investigations, treatments, and surgeries [13,14].

There are many etiologies of back pain; however, most patients seen in primary care will have nonspecific back pain. This is defined as the absence of a direct underlying condition causing back pain and will often spontaneously resolve within a few weeks [15]. While most instances of nonspecific back pain imaging do not necessarily enhance outcomes, diagnostic imaging is frequently utilized during initial assessments. However, current clinical guidelines advocate against such a practice [16-18]. The persistence of overreliance on imaging is multifaceted and not entirely clear. Current literature theorizes that patients' preference for a conclusive diagnosis may drive this behavior [16,18-20]. Both clinicians and patients may perceive diagnostic imaging as crucial for the diagnosis of lower back pain, especially because most patients in acute pain seek a definitive source [16,17]. This perception is further exacerbated by patients overvaluing the role of imaging, which compels clinicians to order imaging to satisfy patient expectations and to avoid potential oversight [13-15,19].

Certain clinical presentations indicate a need for diagnostic imaging. These presentations, often referred to as “red flags” in various clinical guidelines, include factors such as a history of cancer, advanced age, prolonged corticosteroid usage, and severe trauma [15,21]. Symptoms indicative of spinal cord compression, such as difficulties in walking, progressive weakness below the lesion level, accompanying sensory loss, and bladder dysfunction, are particularly concerning. Neurological deficits that raise suspicion for conditions such as cauda equina syndrome and cord compression necessitate further investigation via contrast-enhanced MRI [22]. Early diagnosis of spinal cord compression significantly enhances the likelihood of more favorable clinical outcomes [23]. Conversely, nonambulatory patients prior to diagnosis generally have a diminished prospect of functional recovery following treatment [24].

Diagnostic imaging

Our patient's extensive history of persistent back pain combined with functional ambulatory impairment provided an indication for the urgent use of contrast-enhanced MRI to investigate potential spinal pathology. This imaging modality revealed the presence of spinal compression attributable to an epidural lesion. Notably, axial MRI sections indicated internal flow voids, implying vascular involvement, and thereby heightening the suspicion of a neoplastic etiology for spinal cord compression. Both benign and malignant tumors are known to induce myelopathy due to spinal cord compression. Spinal epidural metastasis often represents an initial sign of malignancy reflecting the propensity of bone as a common metastatic site due to its rich vascular nature [25,26]. However, subsequent analysis of tissue samples obtained from the excised epidural lesion confirmed a non-neoplastic character.

Non-neoplastic causes of spinal cord compression generally include trauma and infectious factors, though traumatic etiologies are not within the scope of this discussion. A noteworthy infectious cause is spinal epidural abscesses. Typically, the clinical presentation of abscesses begins with nonspecific symptoms like fever and malaise that accompany back pain, which may progress over time to radicular pain and neurologic deficits [3,4]. In the present case, the patient's tissue samples exhibited a substantial lymphohistiocytic infiltrate with acute inflammation, initially raising suspicions of infectious etiology or Langerhans cell histiocytosis. However, the absence of fever and malaise lowered infectious etiology on the differential. Further histological examination ruled out the presence of neoplastic elements and returned negative results for CD1a and langerin markers, conclusively ruling out Langerhans cell histiocytosis as a diagnosis. 

Langerhans cell histiocytosis is an uncommon condition characterized by the aberrant proliferation of antigen-presenting macrophages. These cells are predominantly located in the skin and lymphoid tissues. This disorder manifests in a variety of tissue types, with the lungs and bones being the most frequently affected sites [27]. While spinal involvement is more typical in pediatric populations, it remains rare among adults. Differentiating Langerhans cell histiocytosis from other spinal lesions such as infections, lymphomas, and metastases can be challenging; however, a pathologic examination usually reveals a mixed inflammatory infiltrate comprising neutrophils, eosinophils, plasma cells, and lymphocytes. Immunohistochemical staining often yields positive results for CD1a, langerin, S100, and CD68 [27].

Initially, Langerhans cell histiocytosis was considered a potential diagnosis based on MRI findings and the patient's clinical symptoms. Typically, patients with this condition exhibit pain, limited mobility, and sensorimotor impairments, mirroring the presentation of our patient. Additionally, there is a noted propensity for Langerhans cell histiocytosis to affect the thoracic and lumbar vertebral bodies, potentially leading to spinal cord compression due to mass effect [28]. Irrespective of the underlying cause, patients displaying clear symptoms of spinal cord compression necessitate urgent surgical intervention. This approach augments the likelihood of neurological recovery and facilitates histopathological verification [28]. In our case, the patient showed postoperative improvement, yet the histopathological analysis further complicated the understanding of the mass's origin.

Although the definitive etiology of the mass leading to spinal cord compression remains unknown, it exemplifies the diverse and multifaceted origins that can precipitate such a condition. The unusual nature of this non-neoplastic lesion reinforces the understanding that a wide array of spinal structural alterations are capable of creating a mass effect, potentially resulting in spinal cord compression. The findings in this report underscore the importance of considering both neoplastic and non-neoplastic causes in the differential diagnosis of spinal cord compression, thereby enhancing clinical suspicion and improving patient outcomes for underlying spinal conditions.

## Conclusions

Spinal cord compression, a complex neurosurgical concern, manifests with back pain and various neurological deficits. While nonspecific back pain often doesn't warrant immediate imaging, neurological signs amid back pain necessitate radiologic investigation. The spinal cord's vulnerability to various pathologies, including trauma, neoplasms, and infections, underscores the intricate nature of this disorder. In this report, we delineate an uncommon case of a 31-year-old male with chronic back pain, neurological deficits, ataxia, and bladder dysfunction. Contrasting MRI indicated a possible neoplastic etiology, but pathological assessment revealed an unusual non-neoplastic mass suggestive of Langerhans cell histiocytosis or infection. Further scrutiny at Mayo Clinic Laboratories disproved neoplasia and negated Langerhans cell histiocytosis markers. This atypical non-neoplastic lesion exemplifies the diverse spectrum of pathologies inducing spinal cord compression. Emphasizing the consideration of both neoplastic and non-neoplastic causes in diagnosis enhances clinical acumen and augments patient outcomes. Such comprehensive assessment and timely intervention are pivotal in managing spinal conditions, necessitating a holistic approach for accurate diagnosis, effective treatments, and improved patient care.
